# Hyperglycemia promotes myocardial dysfunction via the ERS-MAPK10 signaling pathway in db/db mice

**DOI:** 10.1038/s41374-022-00819-2

**Published:** 2022-08-08

**Authors:** Ya-Wen Deng, Fei Liu, Zhi-Tong Li, Jing-Han Gao, Yong Zhao, Xiao-Lei Yang, Yun-Long Xia

**Affiliations:** Department of Cardiology, Institute of Cardiovascular Diseases, First Affiliated Hospital of Dalian Medical University, No.193, Lianhe Road, Xigang District, 116011 Dalian, China

**Keywords:** Cardiac hypertrophy, Cardiac hypertrophy

## Abstract

Recent studies have demonstrated that hyperglycemia is a major risk factor for the development and exacerbation of cardiovascular disease (CVD). However, the molecular mechanisms involved in diabetic cardiomyopathy (DCM) have not been fully elucidated. In this study, we focused on the underlying mechanism of DCM. Leptin receptor-deficient db/db mice were used to model a type 2 diabetes mellitus (T2DM) model in our study. WT mice and db/db mice received 4-phenylbutyric acid (4-PBA) (25 mg/kg/day) and saline by intraperitoneal injection every other day for 4 weeks. WT and db/db mice were given tail vein injections of 100 μL of rAAV9-Sh-MAPK10 and rAAV9-Sh-GFP at the age of 6–8 weeks. Echocardiography was performed to measure cardiac function, histological examinations were used to evaluate ventricular hypertrophy and fibrosis. Quantitative RT–qPCR was used to assess the mRNA expression of Jun N-terminal kinase 3 (JNK3, MAPK10), atrial natriuretic factor (ANF), brain natriuretic peptide (BNP), and collagen I and III. Immunoblotting was performed to measure the levels of cardiac hypertrophy-related proteins, fibrosis-related proteins, endoplasmic reticulum stress (ERS)-related proteins and apoptosis-related proteins. TUNEL staining was performed to examine cardiomyocyte apoptosis. In contrast to 12-week-old db/db mice, 16-week-old db/db mice showed the most severe myocardial dysfunction. The DCM induced by hyperglycemia was largely alleviated by 4-PBA (25 mg/kg/day, intraperitoneal injection). Similarly, tail vein injection of rAAV9-Sh-MAPK10 reversed the phenotype of the heart in db/db mice including cardiac hypertrophy and apoptosis in db/db mice. The mechanistic findings suggested that hyperglycemia initiated the ERS response through the negative regulation of sirtuin 1 (SIRT1), leading to the occurrence of myocardial dysfunction, and specific knockdown of MAPK10 in the heart directly reversed myocardial dysfunction induced by hyperglycemia. We demonstrated that hyperglycemia promotes DCM in db/db mice through the ERS-MAPK10 signaling pathway in diabetic mice.

## Abstract (web summary)

Hyperglycemia induces endoplasmic reticulum stress and increases expression of MAPK10, and 4-phenylbutyric treatment and the knockdown of MAPK10 both alleviate hyperglycaemia-induced myocardial dysfunction, respectively. Therefore, the present study sheds new light on the novel role of MAPK10 in hyperglycemia-induced myocardial dysfunction in db/db mice.

## Introduction

Cases of diabetes have dramatically increased worldwide, and diabetes mellitus has gradually proven to be a major risk factor for the development and exacerbation of cardiovascular disease (CVD)^1–3^. Independent of epicardial coronary artery disease, hypertension, and valvular disease, diabetic cardiomyopathy (DCM) manifests as the deterioration of longitudinal myocardial dysfunction that is mainly reflected in cardiac hypertrophy^4–6^. Cardiomyocytes enlarge in size, and the ventricular wall becomes thicker to overcome ventricular wall stress and maintain the function and efficiency of the heart in response to increased pressure overload and pathological stimuli; this process is defined as cardiac hypertrophy^7^. Cardiac hypertrophy has been classified as physiological or pathological cardiac hypertrophy, and these forms seem to depend upon the type, duration, and magnitude of deleterious stimuli placed upon the heart^8^. Pathological cardiac hypertrophy and diastolic dysfunction of the ventricle will ultimately progress to severe CVD, including heart failure, arrhythmia, and death^9^.

Perturbations in endoplasmic reticulum (ER) function are referred to as ER stress (ERS), which leads to the accumulation of unfolded and misfolded proteins in the ER and triggers the unfolded protein response (UPR)^10^. Numerous studies have shown the upregulation of UPR pathway-related proteins, such as PERK, activating transcription factor 6, p-EIf2, CHOP, activating transcription factor 4 (ATF4), and IRE1α, in the heart in diabetes models^11–13^. When ERS is prolonged or severe, it induces apoptosis to eliminate unhealthy cells and contributes to cardiac hypertrophy^12,14^. Studies have suggested that the c-Jun NH2-terminal kinase (JNK) pathway is involved in survival signaling, cell death, cancer and diabetes^15^. However, the underlying mechanism of DCM remains unclear, and elucidating the underlying mechanisms of hyperglycemia-induced DCM is vital for the treatment of adverse cardiovascular events in diabetes patients.

In this study, we explored the myocardial functions of db/db mice at different time points. We also observed the attenuation of myocardial dysfunction in db/db mice after treatment with the ERS inhibitor 4-PBA. Moreover, we found that myocardial-specific knockdown of MAPK10 in the heart reversed myocardial dysfunction in diabetic mice. Our findings suggested that MAPK10 is a key molecule in hyperglycemia-induced cardiac remodeling.

## Materials and methods

### Ethics statements, animals and treatment

Six-eight-week-old male mice, including wild-type (WT) and BKS-Lepr^em2Cd479^/Gpt (db/db) mice, were purchased from GemPharmatech Co. Ltd. All mice were maintained in the mouse barrier facilities of Dalian Medical University under 12 h light/dark cycles with free access to a normal diet and water. We measured blood glucose every week with a glucometer (PHI8080005) (Beijing, China) using the glucose oxidase method and glucose strips (DP0LM3F03A) (Beijing, China). At the age of 12 weeks, WT and db/db mice were intraperitoneally injected with 4-phenylbutyric acid (4-PBA) (HY-AO281) (25 mg/kg/day), which was purchased from MedChemExpress (Monmouth Junction, NJ, USA), or an equal amount of saline every other day until the hearts were extracted. WT and db/db mice were injected with rAAV9 expressing green fluorescent protein (rAAV9-GFP) or mitogen-activated protein kinase (MAPK) 10-specific short hairpin RNA (rAAV9-Sh-MAPK10) at the age of 6–8 weeks, and these viruses were produced by Hanbio (Shanghai, China) according to the manufacturer’s protocol. All animal experiments complied with the Guidelines of the Institutional Animal Care and Use Committee of Dalian Medical University, who approved all of the protocols.

### Echocardiography

Transthoracic echocardiography was measured using a Vevo 1100 High-Resolution Imaging System (Visual Sonics, Inc, Toronto, Ontario, Canada) as reported previously^16^. Left ventricular (LV) ejection fraction (EF) and fractional shortening (FS) were determined using parasternal short axis M-mode imaging and averaged from three cardiac cycles^16^. Pulse-wave Doppler images of mitral inflow from the apical 4-chamber view were used to determine the transmitral E/A ratio, which is an index of LV diastolic parameters^17^.

### RNA extraction and real-time polymerase chain reaction (RT–PCR)

Total RNA was extracted from mouse hearts with TRIzol reagent (Invitrogen, Carlsbad, CA, USA). RNA (1 μg) was reverse transcribed into cDNA using an RT kit (MedChemExpress, Monmouth Junction, NJ, USA), and the cDNA was subsequently used as a template for quantitative RT–PCR. Quantitative RT–PCR was performed with SYBR Green qPCR reagents (MedChemExpress, Monmouth Junction, NJ, USA), and β-actin was used as an internal control. The following primers were used for PCR analysis. ANF: forward 5′-CTG GGA CCC CTC CGA TAG AT-3′, reverse 5′-TTC GGT ACC GAA GCT GTT G-3′; BNP, forward 5′-TTT GGG CTG TAA CGC ACT GA-3′, reverse 5′-CAC TTC AAA GGT GGT GGT GGT CCC AGA-3′; Collagen I: forward 5′-CCT CAG GGT ATT GCT GGA CAA C-3′, reverse 5′-CAG AAG GAC CTT GTT TGC CAG G-3′; Collagen III: forward 5′-TGA CTG TCC CAC GTA AGC AC-3′, reverse 5′-GAG GGC CAT AGC TGA ACT GA′; MAPK10: forward 5′-AGG TGG ACA ACC AGT TCT ACA-3′, reverse 5′-GCA CAG ACT ATT CCC TGA GCC-3′, β-actin: forward 5′-ACT GCC GCA TCC TCT TCC T-3′, reverse 5′-TCA ACG TCA CAC TTC ATG ATG ATG GA-3′ and collagen III: forward 5′-AAA TTC TGC CAC CCC GAA CT-3′, reverse: 5′-CCA GTG CTT ACG TGG GAC AGT-3′.

### Western blot analysis

Proteins were extracted from snap-frozen heart tissues using RIPA buffer (Solarbio Science Technology Co, Beijing, China). Protein separation was performed by electrophoresis on 10% SDS–PAGE gels, and the proteins were transferred to polyvinylidene difluoride membranes and incubated with the corresponding antibodies at 4 °C for 1–2 days. Antibodies against IRE1α (#3364EA39) were purchased from Invitrogen (Carlsbad, CA, USA). Antibodies against sirtuin 1 (SIRT1) (#13161-1-AP), Caspase 3 (#19677-1-AP), Bcl2 (#26593-1-AP), Bax (#50599-1-AP) and β-Tubulin (#10094-1-AP) were purchased from Proteintech Group (Rosemont, USA). Antibodies against transforming growth factor-β1 (TGF-β1) (#3711 S), CHOP (#P35638), pERK1/2 (#P27361), and ERK1/2 (#P28482) were purchased from Cell Signaling Technology (Boston, MA, USA). Antibodies against ATF4 (#ab23760) and MAPK10 (#ab126591) were purchased from Abcam (London, England). Then, the membranes were incubated with horseradish peroxidase-conjugated secondary antibodies (1:3000–1:5000) for at least 1 h at room temperature. All blots were developed using the ECL Plus chemiluminescence system, and signal intensities were analyzed with a Gel-Pro 4.5 Analyzer (Media Cybernetics, USA).

### Histopathological analysis

Mice were euthanized and flushed with physiological saline through the left ventricle. The hearts were extracted and weighed, and the lengths of the tibiae were also measured. The upper 2/3 of the heart was fixed with 4% paraformaldehyde for ~48 h, embedded in paraffin and sectioned (4 μm). Ventricular sections were stained with hematoxylin and eosin (H&E) (G1120; Solarbio, Beijing, China), Masson’s trichrome (G1340; Solarbio, Beijing, China), and rhodamine-labeled wheat germ agglutinin (WGA) (1.25 mg/mL; ZD0510, Vector Laboratory, Burlingame, CA, USA). The images of Masson’s trichrome-stained ventricular sections taken at ×100 magnification were evaluated by a pathologist in a double-blinded manner and were analyzed using an 11 mega pixel CCD camera (Olympus-SIS, Shinjuku, Tokyo, Japan). The positive areas were quantified performed with NIH Image 1.61 software.

### TUNEL assay

Cardiomyocyte apoptosis in heart sections was assessed using a TDT-mediated dUTP nick end labeling (TUNEL) system (Roche, Mannheim, Germany) according to the manufacturer’s instructions. Cardiomyocytes were first identified by immunohistochemical staining with α-actinin (6487 T), which was purchased from Cell Signaling Technology (Boston, MA, USA). Then, cardiomyocytes were stained with TUNEL staining dye, and the nuclei were counterstained with DAPI. The number of TUNEL-positive cardiomyocytes was counted in 10 fields per section under a microscope, and the percentage of TUNEL-positive cardiomyocytes was determined.

### Statistical analysis

The results are presented as means ± standard deviation (SD). Differences between groups were analyzed using two-way ANOVA with GraphPad Prism 8 software, and a *p* value < 0.05 was considered statistically significant.

## Results

### Hyperglycemia contributes to cardiac hypertrophy and diastolic dysfunction in db/db mice

**Fig. 1:**
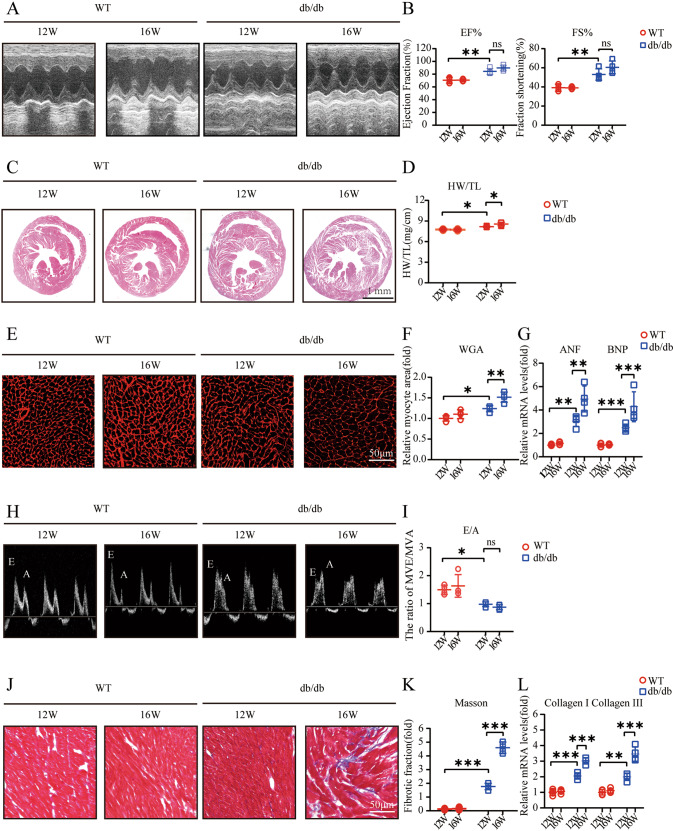
Hyperglycemia contributes to cardiac hypertrophy and diastolic dysfunction in db/db mice. **A** M-mode echocardiography of left ventricular chamber; **B** Measurements of ejection fraction (EF%) and fractional shortening (FS%) at 12 and 16 weeks of age in mice (*n* = 4); **C** Hematoxylin and eosin (H&E) staining of heart sections (left) at 12 and 16 weeks of age in mice; **D** The heart weight to tibial length (HW/TL) ratio (*n* = 4); **E**, **F** TRITC-labeled wheat germ agglutinin (WGA) staining of heart sections and quantification of myocyte cross-sectional area (200 cells counted per heart, *n* = 4) at 12 and 16 weeks of age in mice; **G** Quantitative real-time polymerase chain reaction (PCR) analysis of hypertrophic genes including atrial natriuretic factor (ANF) and brain natriuretic peptide (BNP) at 12 and 16 weeks of age in mice (*n* = 4); **H**, **I** Pulse-wave Doppler images of mitral inflow from the apical 4-chamber (left), and calculation of E/A ratio of 12-week-old and 16-week-old mice (*n* = 4); **J** and **K** Masson’s trichrome staining of cardiac interstitial fibrosis and quantification of fibrotic area (*n* = 4); **L** Relative mRNA levels of interstitial fibrosis-associated genes (Collagen I and III) of mouse hearts (*n* = 4).

To explore whether hyperglycemia leads to myocardial dysfunction, we first performed echocardiography, and the echocardiography assessments demonstrated an increase in LV contractile function, which was mainly reflected by increases in the EF% and FS%. Sixteen-week-old db/db mice showed mild increases in heart contractility compared to 12-week-old db/db mice because 12-week-old db/db mice already showed extensive increases in cardiac contractility (Fig. 1A, B). In addition, 16-week-old db/db mice showed a modest increase in the heart weight/tibia length (HW/TL) ratio in contrast to 12-week-old db/db mice (Fig. 1C, D). Similarly, the myocyte cross-sectional area of db/db mice was dramatically increased compared with that of WT mice, and 16-week-old db/db mice were larger than 12-week-old db/db mice (Fig. 1E, F). Moreover, the indicator of ventricular diastolic function, the transmitral E/A ratio, decreased in db/db mice specifically, 16-week-old db/db mice showed the lowest E/A ratios (Fig. 1H, I). Hypertrophy-associated genes, including atrial natriuretic factor (ANF) and brain natriuretic peptide (BNP), exhibited elevated mRNA expression levels in db/db mice, especially in 16-week-old db/db mice (Fig. 1G). Cardiac fibrosis was demonstrated by the upregulation of fibrosis-related genes, including collagen I and collagen III, which are downstream of TGF-β1, and a significant increase in the interstitial fibrosis area fraction (Fig. 1J–L).

### PBA reverses hyperglycemia-induced cardiac hypertrophy and diastolic dysfunction in db/db mice

**Fig. 2:**
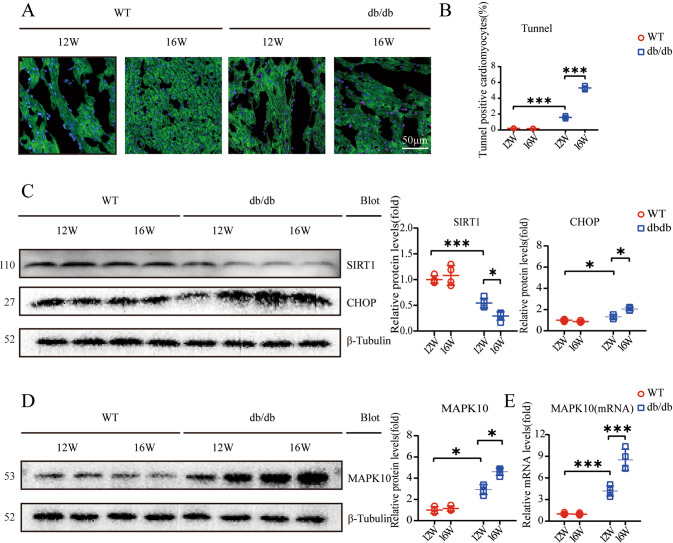
Hyperglycemia induces ERS, apoptosis, and a conspicuous change in the expression of MPAK10 and SIRT1. **A**, **B** Expression of TUNEL-positive cardiomyocytes in heart sections (*n* = 4). Scale bar: 50 μm; **C** Relative protein levels of SIRT1 and CHOP, β-Tubulin was used as an internal control (*n* = 4); **D** Relative Protein levels of MAPK10, β-Tubulin was as an internal in protein quantification (*n* = 4); **E** Relative mRNA levels of MAPK10, and β-actin was used as an internal control for mRNA quantification (*n* = 4).

**Fig. 3:**
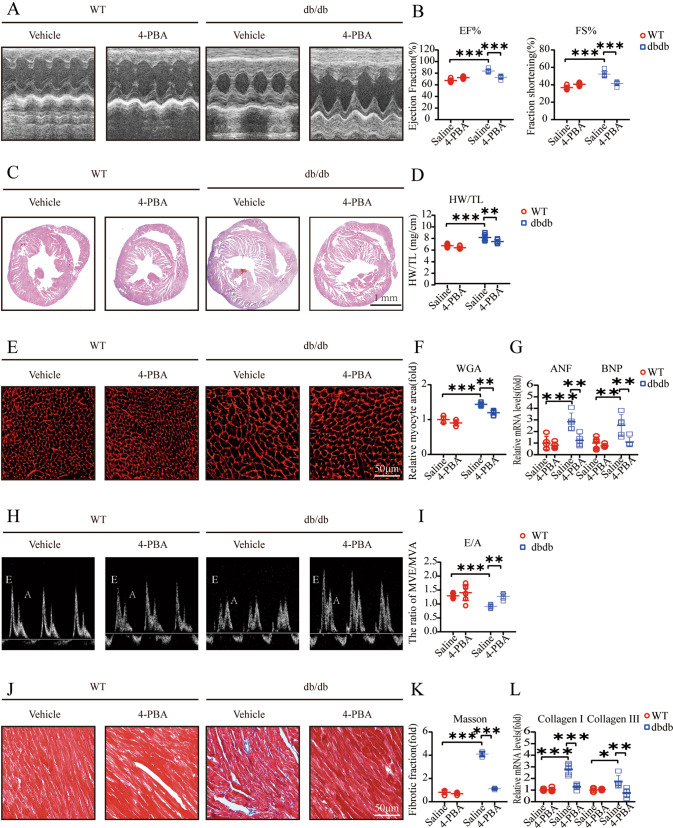
4-PBA attenuates hyperglycemia-induced cardiac hypertrophy and diastolic dysfunction in db/db mice. **A** M-mode echocardiography of the left ventricular chamber; **B** Measurements of ejection fraction (EF%) and fractional shortening (FS%) (*n* = 7); **C** Hematoxylin and eosin (H&E) staining of heart sections (left); **D** The heart weight to tibial length (HW/TL) ratio (*n* = 7); **E**, **F** TRITC-labeled wheat germ agglutinin (WGA) staining of heart sections (left) and quantification of myocyte cross-sectional area (200 cells counted per heart, *n* = 7); **G** Quantitative real-time polymerase chain reaction analysis of hypertrophic genes, including atrial natriuretic factor (ANF) and brain natriuretic peptide (BNP) (*n* = 7); **H**, **I** Pulse-wave Doppler images of mitral inflow from the apical 4-chamber, and calculation of E/A ratio in WT and db/db (*n* = 7); **J**, **K** Masson’s trichrome staining of cardiac interstitial fibrosis. Quantification of fibrotic area (*n* = 7); **L** Relative mRNA levels of interstitial fibrosis-associated genes (collagen I and III) of mouse hearts (*n* = 7).

Hyperglycemia might induce the ERS response^18^ and cardiomyocyte apoptosis^19^. As an important factor regulating ERS, CHOP was strongly upregulated and was relatively high in 16-week-old db/db mice compared with 12-week-old db/db mice (Fig. 2C). Furthermore, TUNEL staining showed an increase in the apoptosis-positive fraction (Fig. 2A, B). Moreover, it was previously reported that hyperglycemia inhibits SIRT1 expression^20^, and SIRT1 deficiency exacerbates ERS-related CVD^21^. In our study, we demonstrated a decrease in SIRT1 expression, especially in 16-week-old db/db mice (Fig. 2C). In addition, MAPK10 protein and mRNA expression was increased (Fig. 2D, F). 4-PBA is an inhibitor of ERS^22^. To explore whether 4-PBA alleviates cardiac dysfunction in db/db mice, WT and db/db mice were treated with 4-PBA (25 mg/kg/day) by intraperitoneal injection beginning at 12 weeks of age. It was demonstrated that 4-PBA improved myocardial contractility, maintaining relatively lower levels of EF% and FS% (Fig. 3A, B). In addition, hypertrophy markers, including the HW/TL ratio, the cross-sectional area of cardiomyocytes and the mRNA expression levels of ANF and BNP, were dramatically decreased (Fig. 3C–G). The transmitral E/A ratio was elevated in db/db mice after treatment with 4-PBA (Fig. 3H, I). Masson staining showed a decreased fibrotic fraction, and quantitative RT–PCR showed downregulated fibrotic gene expression after the administration of 4-PBA (Fig. 3J–L).

### 4-PBA attenuated ERS and apoptosis and further exacerbates cardiac dysfunction

**Fig. 4:**
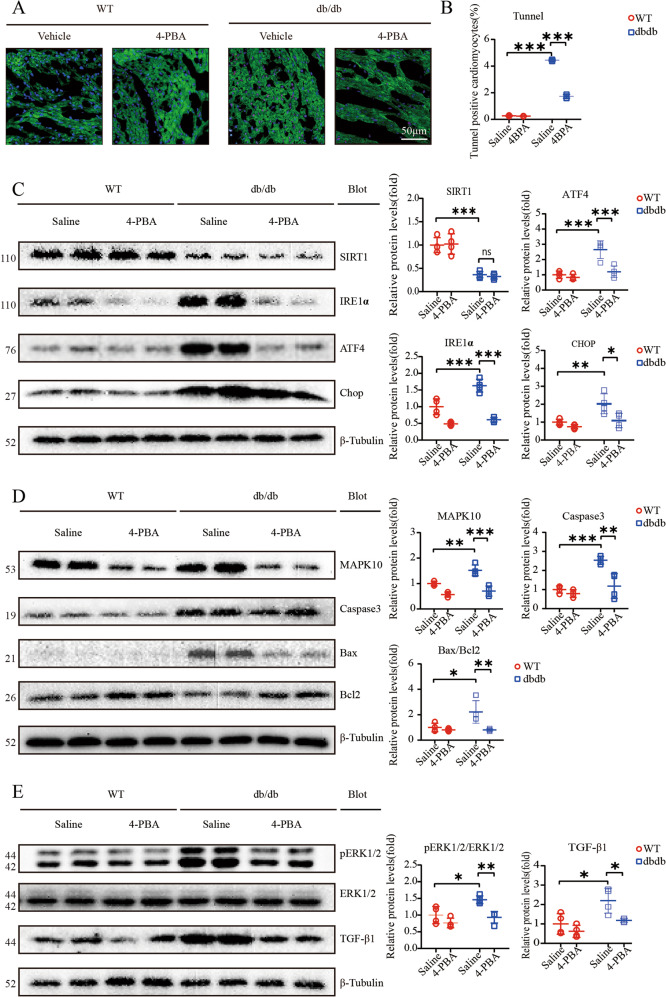
4-PBA attenuates hyperglycemia-induced myocardial dysfunction. **A**, **B** Expression of TUNEL-positive cardiomyocytes in heart sections (*n* = 4). Scale bar: 50 μm; **C** Relative levels of endoplasmic reticulum stress (ERS)-associated proteins including ATF4, IRE1α, and CHOP; β-tubulin was used as an internal control (*n* = 4); **D** Relative protein levels of MAPK10, and apoptosis-associated proteins, including Caspase3 and Bax/Bcl2, β-Tubulin was as an internal (*n* = 4); **E** Immunoblotting analysis of pERK12/ERK1/2 and TGF-β1 protein levels in the hearts and quantification (*n* = 4).

Our study suggested that the expression of SIRT1 was not altered by 4-PBA; however, we measured ERS pathway-associated proteins by western blotting, and the results suggested that IRE1α, ATF4, and CHOP expression was robustly reduced (Fig. 4C). TUNEL staining indicated that 4-PBA largely decreased the fraction of cardiomyocyte apoptosis in db/db mice (Fig. 4A, B). Apoptosis-related pathway proteins, including Caspase3 and Bax/Bcl2, were significantly downregulated after 4-PBA treatment (Fig. 4D). Furthermore, MAPK10 protein expression was strongly decreased by 4-PBA (Fig. 4D). TGF-β signaling is an important pathway in cardiac fibrosis, and our data indicated that 4-PBA strongly reduced TGF-β protein expression; furthermore, the enhanced phosphorylation of the hypertrophy marker ERK1/2 was markedly decreased (Fig. 4E).

### Knockdown of MAPK10 in db/db mouse hearts reverses cardiac hypertrophy and diastolic dysfunction

**Fig. 5:**
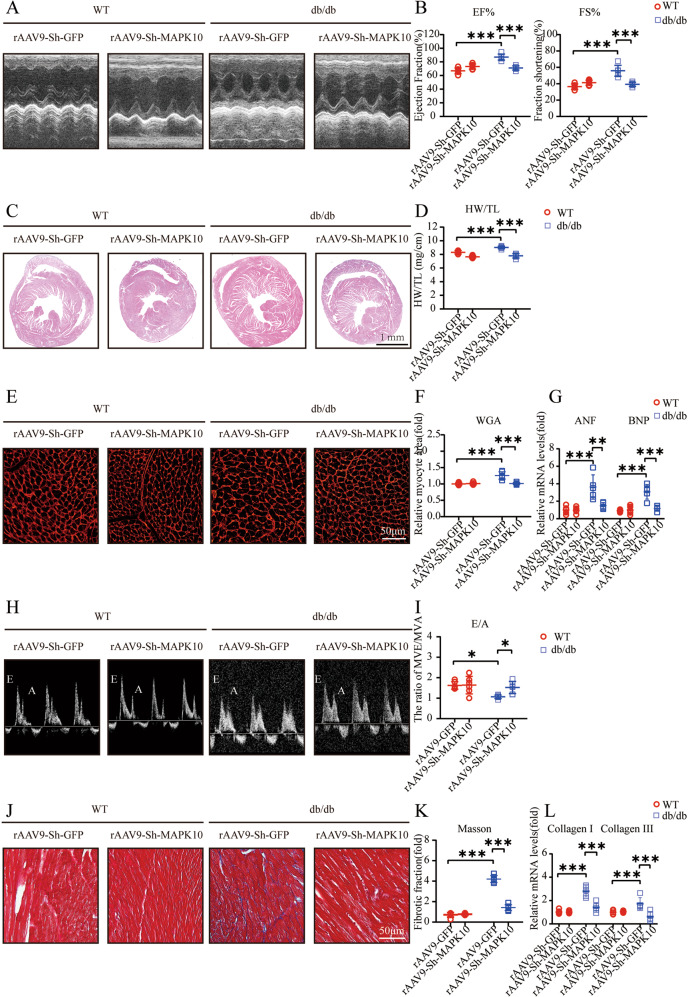
The knockdown of MAPK10 reverses cardiac hypertrophy and diastolic dysfunction. **A**, **B** M-mode echocardiography of left ventricular chamber, and measurements of ejection fraction (EF%) and fractional shortening (FS%) (*n* = 6); **C** Hematoxylin and eosin (H&E) staining of heart sections (left); **D** The heart weight to tibial length (HW/TL) ratio (*n* = 6); **E**, **F** TRITC-labeled wheat germ agglutinin (WGA) staining of heart sections (left) and quantification of myocyte cross-sectional area (200 cells counted per heart, *n* = 6); **G** Quantitative real-time polymerase chain reaction analysis of hypertrophic genes, including atrial natriuretic factor (ANF) and brain natriuretic peptide (BNP) (*n* = 6); **H**, **I** Pulse-wave Doppler images of mitral inflow from the apical 4-chamber and calculation of E/A ratio in WT and db/db (*n* = 6); **J**, **K** Masson’s trichrome staining of cardiac interstitial fibrosis. Quantification of fibrotic area (*n* = 6); **L** Relative mRNA levels of interstitial fibrosis-associated genes (collagen I and III) in mouse hearts (*n* = 6).

We further examined whether MAPK10 knockdown specifically in mouse hearts could reverse cardiac dysfunction in db/db mice. Cardiac contractility in rAAV9-sh-MAPK10-injected db/db mice was improved, as reflected by the decreases in EF% and FS% (Fig. 5A, B). In addition, hypertrophy markers were significantly reduced after MAPK10 knockdown (Fig. 5E–G). The transmitral E/A ratio increased considerably (Fig. 5H, I). Moreover, the interstitial fibrotic fraction and expression of fibrosis-related genes, including collagen I and III, decreased dramatically. (Fig. 5J–L).

### Knockdown of MAPK10 alleviates apoptosis and myocardial dysfunction

**Fig. 6:**
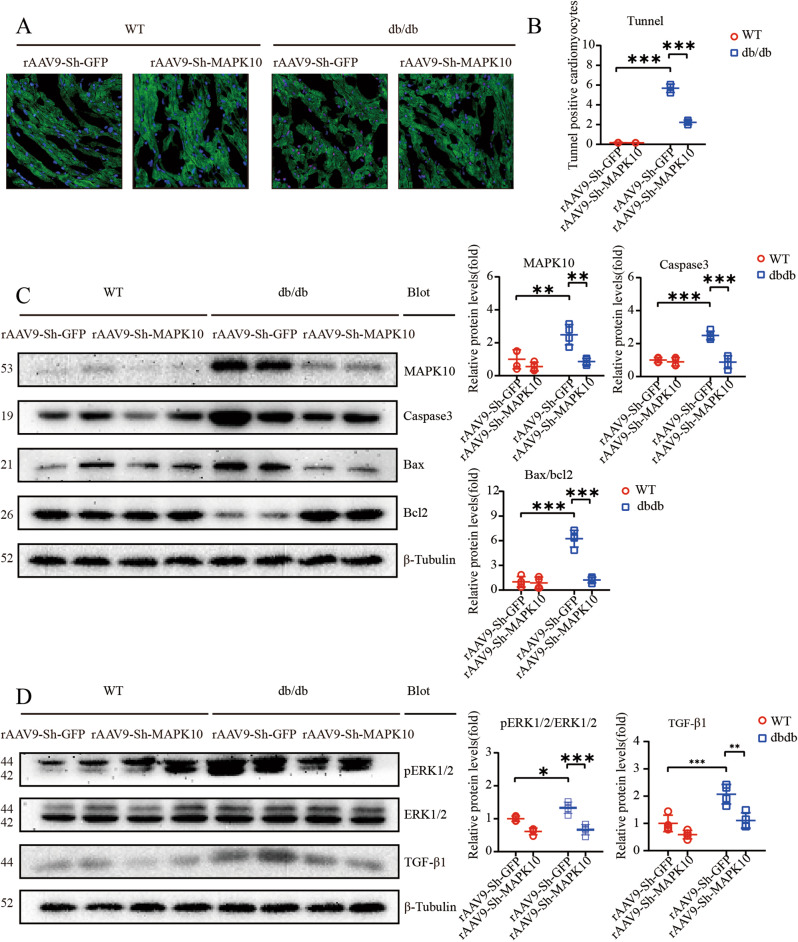
The knockdown of MAPK10 alleviates apoptosis and cardiac dysfunction. **A**, **B** Expression of TUNEL-positive cardiomyocytes in heart sections (*n* = 4). Scale bar: 50 μm; **C** Relative protein levels of MAPK10, and apoptosis-associated protein expression inclusive of cleaved Caspase3 and Bax/Bcl2. β-Tubulin was used as an internal control (*n* = 4); **D** Immunoblotting analysis of pERK12/ERK1/2 and TGF-β1 protein levels in the hearts and quantification (*n* = 4).

The rAAV9-sh-MAPK10-mediated decrease in MAPK10 levels was evident by western blotting, and MAPK10 protein levels were notably decreased (Fig. 6C). TUNEL staining showed a sharp reduction in the TUNEL-positive cardiomyocyte fraction in rAAV9-sh-MAPK10-injected db/db mouse hearts (Fig. 6A, B). In addition, we demonstrated significant reductions in Caspase3 and Bax/Bcl2 protein levels (Fig. 6C). Western blotting showed a distinct decrease in the protein expression of p-ERK1/2, ERK1/2 and TGF-β1, which was associated with the alleviation of cardiac hypertrophy and fibrosis (Fig. 6D).

## Discussion

**Fig. 7:**
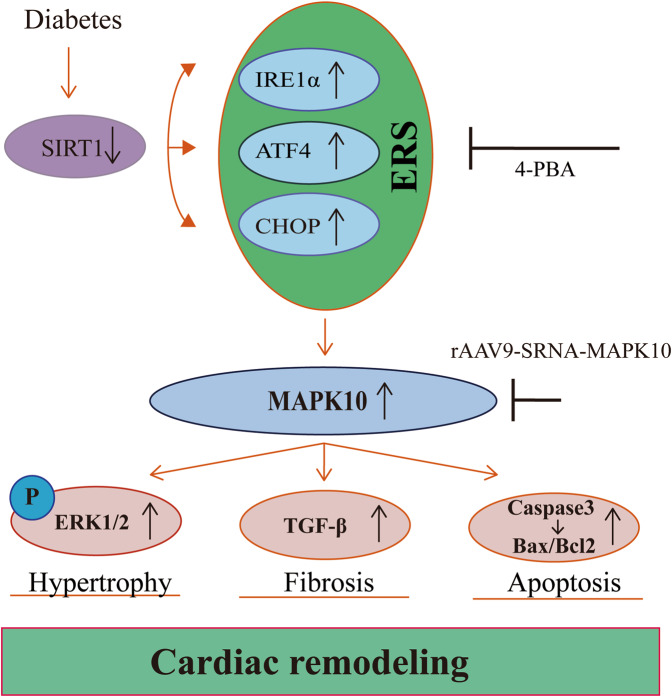
A working model of hyperglycemia in DCM. Hyperglycemia leads to the reduced expression of SIRT1, and the downregulated SIRT1 is responsible for the activation of ERS. Meanwhile, ERS activation stimulates the overexpression of MAPK10 at the mRNA and protein levels, which is a key event in hyperglycemia-induced cardiac remodeling.

The present study identified a novel physiological role of MAPK10 in reversing ERS-induced DCM (Fig. 7). Twelve-week-old db/db mice began to exhibit mild DCM, while 16-week-old db/db mice exhibited more obvious systolic dysfunction, diastolic dysfunction and apoptosis, and MAPK10 expression increased with time. The ERS inhibitor 4-PBA significantly attenuated the expression of MAPK10 and alleviated DCM. Similarly, MAPK10 knockdown in db/db mouse hearts strongly reversed the deleterious effects, including hyperglycemia-induced cardiac hypertrophy, diastolic dysfunction and apoptosis. Thus, MAPK10 is a crucial factor in hyperglycemia-induced DCM. The working model of hyperglycemia in DCM is illustrated in Fig. 7.

Previous data have shown that cardiomyocytes and adult-inducible SIRT1-knockout mice are vulnerable to cardiac injury^21^, and SIRT1 activation was demonstrated to be a therapeutic target for DCM^23,24^. Mechanistically, SIRT1 is responsible for the activation of CHOP in DCM in vitro^25^. Our findings suggested that SIRT1 protein expression is significantly decreased, especially in 16-week-old db/db mice (Fig. 2C). We also showed an increase in CHOP protein levels, indicating that the ERS signaling pathway was activated (Fig. 2C). The key features of ERS signal transduction are increased expression of the ERS sensors IRE1α, ATF4 and CHOP^26,27^. Evidence also suggests that the translation of ATF4 activates the expression of CHOP by directly binding to its 5′-UTR of CHOP^27^. Therefore, we used 4-PBA to explore the role of the ERS response by measuring the expression levels of these proteins. The results showed that treatment with 4-PBA inhibited the expression of IRE1α, ATF4, and CHOP, suggesting that the ERS response is attenuated by 4-PBA (Fig. 4C). Moreover, db/db mice began to exhibit ventricular hypertrophy at 12 weeks, and the most severe systolic dysfunction was observed at 16 weeks, which mainly manifested as elevated EF% and FS% and increases in other hypertrophy markers, including the HW/TL ratio and cross-sectional myocardial area (Fig. 1E–G). The results also showed diastolic dysfunction in db/db mice, which manifested as a decrease in the E/A ratio and increased interstitial fibrosis (Fig. 1H–L). Our data also demonstrated apoptosis in 16-week-old db/db mice (Fig. 2A, B). The use of 4-PBA was associated with the alleviation of myocardial dysfunction in db/db mice (Fig. 3). Surprisingly, MAPK10 protein expression also showed evident decreases with time in db/db mice (Fig. 4).

Activation of the ERS response tends to be associated with the pathophysiological processes of multiple CVDs. It has been shown that ERS activation leads to the activation of numerous pathways, such as fibrosis, inflammation and hypertrophy. And Zhang et al.^28^ concluded that the activation of ERS, manifested in the upregulated expression of CHOP, was responsible for the stimulation of the MAPK signaling pathway, which ultimately led to heart failure^28^. It was demonstrated that the activation of ERS might enhance the phosphorylation of ERK1/2, as well as the activation of TGF-β1, which in turn can accelerate the occurrence and development of myocardial remodeling^24^. Therefore, the use of ERS inhibitors in db/db mice might not demonstrate whether downstream MAPK10 plays a key role in DCM. MAPK10 (JNK3) is a member of the JNK family, which includes JNK1, JNK2, and JNK3^29^, and was reported to be mainly expressed in the heart, brain, and testicles, playing a central role in the ERS response^30^. More importantly, the basal activity of JNK3 is relatively low; under stress, JNK3 is activated through upregulation^31^. Previous data also showed that MAPK10 is a key molecule associated with apoptosis in cardiomyocytes, and IRE1α is responsible for the activation of JNK^32^. We therefore performed heart-specific knockdown of MAPK10 to verify whether MAPK10 is a key molecule in hyperglycemia-induced cardiac remodeling. The data showed that myocardial dysfunction, including systolic dysfunction, diastolic function, and apoptosis in db/db mice, was completely reversed in db/db mice via the TGF-β signaling pathway and ERK1/2 phosphorylation (Fig. 6D). Moreover, the expression of the apoptosis-related protein Caspase3 and the Bax/Bcl2 ratio were robustly reduced.

There were several limitations in this study. First, db/db mice are a classic T2DM model of leptin receptor deficiency, which is widely used in basic research. However, leptin receptor deficiency is just one of mechanisms of T2DM, and such a mechanism is rare, while hyperinsulinemia and insulin resistance, caused by other risk factors, including environmental factors, genetics and epigenetics, are more common in human T2DM. Therefore, exploring and verifying the outcomes and mechanisms in more T2DM models are needed in the future. Second, we did not conduct an in vitro experiment to validate the hyperglycemia-induced MAPK10 activation.

In conclusion, this study showed that hyperglycemia promotes myocardial dysfunction in db/db mice through the ERS-MAPK10 signaling pathway. MAPK10 might become a potential target for future cardioprotective therapeutic strategies for diabetes-associated CVDs.

## Supplementary information

Supplementary Table

## Data Availability

The data used to support the findings of this study are available from the corresponding authors upon reasonable request.
