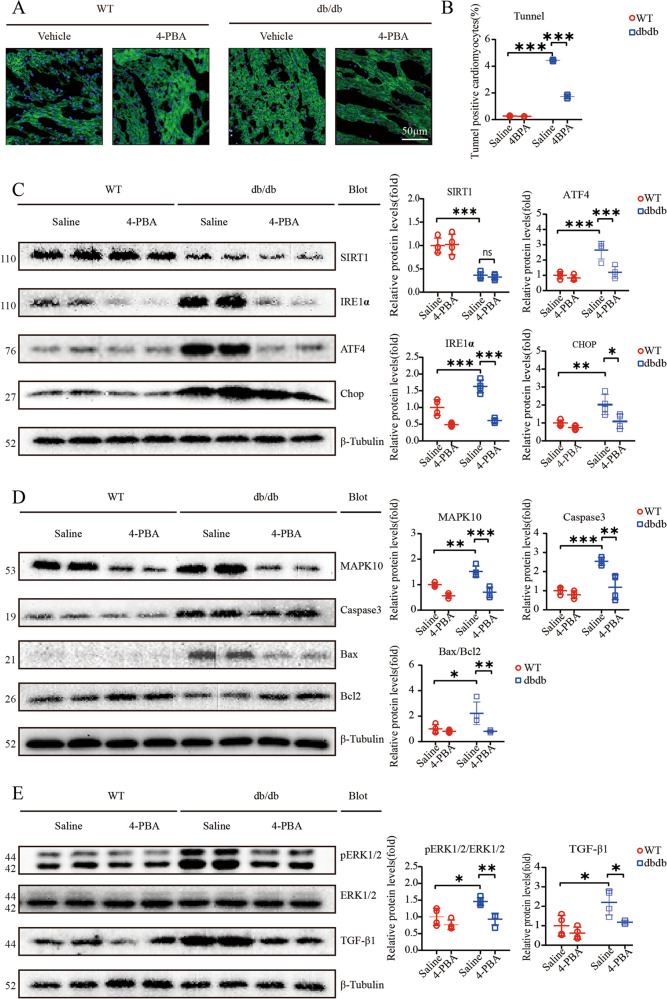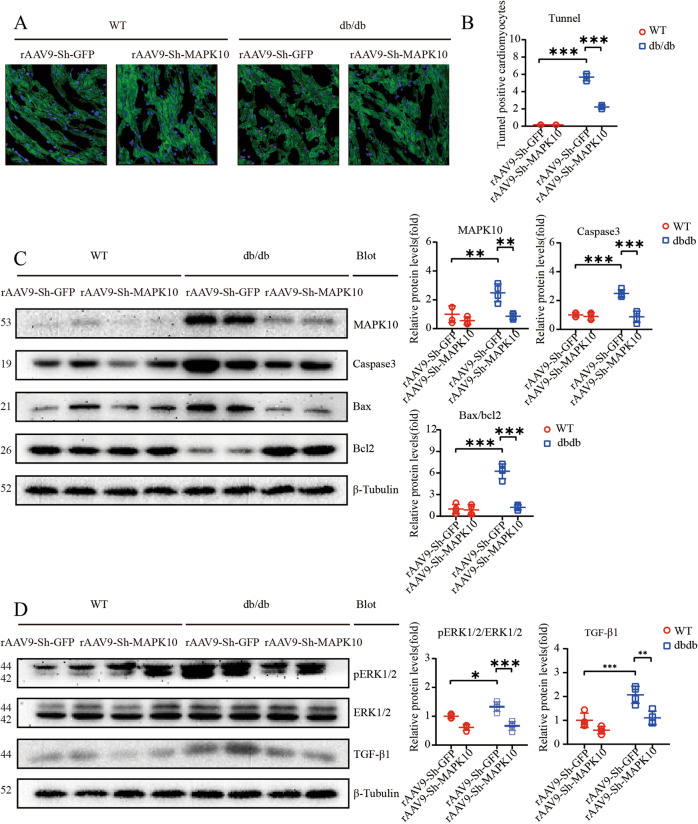# Correction to: Hyperglycemia promotes myocardial dysfunction via the ERS-MAPK10 signaling pathway in db/db mice

**DOI:** 10.1038/s41374-022-00833-4

**Published:** 2022-08-30

**Authors:** Ya-Wen Deng, Fei Liu, Zhi-Tong Li, Jing-Han Gao, Yong Zhao, Xiao-Lei Yang, Yun-Long Xia

**Affiliations:** Department of Cardiology, Institute of Cardiovascular Diseases, First Affiliated Hospital of Dalian Medical University, No.193, Lianhe Road, Xigang District, 116011 Dalian, China

**Keywords:** Cardiac hypertrophy, Cardiac hypertrophy

Correction to: *Laboratory Investigation* 10.1038/s41374-022-00819-2, published online 08 August 2022

The original version of this article unfortunately contained mistakes in Figs. 4 and 6. The authors found that the Tunnel staining of Fig. 4 was the same as Fig. 6. The correct figures can be found below. The original article has been corrected.