# Supplementary material for: Hyperglycemia promotes myocardial dysfunction via the ERS-MAPK10 signaling pathway in db/db mice
Source: Lab Invest. 2022 Aug 8;102(11):1192–202. doi: 10.1038/s41374-022-00819-2 (PMC9588458; doi:10.1038/s41374-022-00819-2)
Supplement: Supplementary file 1 — Supplementary Table [file 41374_2022_819_MOESM1_ESM.docx]

**Supplement**

**Supplementary Table**

**Table1** Record of blood glucose concentration of WT and db/db mice every week since mice were at the age of 9-week

|  | week 9 | week 10 | week 11 | week 12 | week 13 | week 14 | week 15 | week 16 |
| --- | --- | --- | --- | --- | --- | --- | --- | --- |
| WT | 6.2 | 6.7 | 7.4 | 7.8 | 7.7 | 7.6 | 7.4 | 6.5 |
| db/db | 24.5 | 23.3 | 22.1 | 21.9 | 24.8 | 23.9 | 25.5 | 24.1 |
| WT+Saline | 7.5 | 6.7 | 6.9 | 6.9 | 6.8 | 6.6 | 6.5 | 7.9 |
| WT+4-PBA | 6.6 | 7.9 | 6.9 | 6.8 | 6.6 | 7.5 | 7.5 | 6.8 |
| db/db+Saline | 25.2 | 22.1 | 25.5 | 24.9 | 25.3 | 25.4 | 24.3 | 24.7 |
| db/db+4-BPA | 24.7 | 25.2 | 23.8 | 22.8 | 23.5 | 24.2 | 23.9 | 23.9 |
| WT-rAAV-Sh-GFP | 7.6 | 7.4 | 7.7 | 8.2 | 7.1 | 7.6 | 7.9 | 7.3 |
| WT-rAAV-Sh-MAPK10 | 8.4 | 7.4 | 7.5 | 7.8 | 6.8 | 6.6 | 8.3 | 6.8 |
| db/db-rAAV-Sh-GFP | 25.0 | 26.0 | 23.8 | 21.9 | 22.4 | 23.3 | 23.4 | 25.1 |
| db/db-rAAV-Sh-MAPK10 | 24.4 | 24.9 | 23.5 | 23.5 | 24.4 | 23.0 | 24.6 | 24.5 |

Abbreviation: db/db: BKS-Lepr^em2Cd479^/Gpt; 4-BPA: 4-Phenylbutyric; rAAV9-Sh-MAPK10:mitogen-activated protein kinase (MAPK) 10-specific short hairpin RNA; rAAV9-GFP: rAAV9 expressing green fluorescent protein; MAPK10 (JNK3): Jun N terminal kinase 3;
